# Trends and socio-demographic components of modern contraceptive use among sexually active women in Rwanda: a multivariate decomposition analysis

**DOI:** 10.1186/s12978-022-01545-0

**Published:** 2022-12-16

**Authors:** Chester Kalinda, Million Phiri, Kafiswe Chimpinde, Marie C. S. Ishimwe, Simona J. Simona

**Affiliations:** 1 Bill and Joyce Cummings Institute of Global Health, University of Global Health Equity, Kigali Heights, Plot 772 KG 7 Ave., P. O. Box 6955, Kigali, Rwanda; 2 School of Humanities and Social Sciences, University of Zambia, Great East Road Campus, P. O. Box 32379, Lusaka, Zambia; 3 Institute of Global Health Equity Research (IGHER), University of Global Health Equity, Kigali Heights, Plot 772 KG 7 Ave, P. O. Box 6955, Kigali, Rwanda; 4 School of Public Health and Social Sciences, University of the Witwatersrand, Johannesburg, South Africa

**Keywords:** Decomposition analysis, Contraceptives use, Rwanda demographic health surveys

## Abstract

**Background:**

The attainment of targets set for modern contraceptive use remains a challenge in sub-Saharan Africa. Rwanda, in its new Family Planning and Adolescent Sexual Reproductive Health/Family Planning (FP/ASRH) Strategic Plan 2018–2024 has set the attainment of a contraceptive prevalence rate (CPR) of 60% by 2024. To achieve this, identifying factors that enhance modern contraceptive use among sexually active women is critical.

**Methods:**

We used three Rwanda Demographic Health Surveys (RDHS) datasets collected in 2010, 2015, and 2019/2020 in a multivariable decomposition analysis technique to describe trends and identify factors influencing change in modern contraceptive use among sexually active women aged 15–49 years. Results presented as coefficients and percentages took into consideration the complex survey design weighted using StataSE 17.

**Results:**

Modern contraceptive use increased from 40% in 2010 to 52.4% in 2020 among sexually active women. About 23.7% of the overall percentage change in modern contraceptive use was attributable to women’s characteristics which included women’s education levels, number of living children, and being told about family planning at health facilities. Coefficients contributed 76.26% to the change in modern contraceptive use. This change was attributed to modern contraceptive use among young women between the age of 20–24 years, women’s education level, the number of living children, changes in family size, and being visited by community health workers.

**Conclusion:**

Rwanda remains on course to archive its 2024 family planning targets. However, there is a need to enhance programs that target sexually active adolescents and young adults, and women from rural areas to sustain the gains made. Furthermore, continuous support of community health workers will be key in exceeding the set targets of modern contraceptive use among sexually active women in Rwanda.

## Plain Language Summary

Modern contraceptive use has been observed to improve the health of women and accelerate the attainment of health rights and gender equity. Sustaining and enhancing the uptake of modern contraceptives is essential in designing sexual reproductive health programs for all sexually active women to reduce the risks of unplanned pregnancies. This paper identified and reports changes in modern contraceptives among all sexually active women aged between 15 and 49 years old for the period 2010–2020. The manuscript used Rwanda Demographic and Health Survey (RDHS) data that is collected every after five years to track changes in the health and demographics of the citizens. The study found that a change in the behaviour of women towards contraceptive use is key to improving modern contraceptive use among sexually active women. Therefore, focusing on these behavioural components will enhance modern contraceptive use and contribute to improving women’s sexual and reproductive health.

## Introduction

The number of women with met needs for modern contraceptives and the modern contraceptive prevalence rate (mCPR) remain critical indicators in the attainment of health equity and Sustainable Development Goals (SDGs) [1, 2]. Attainment of SDG 3 which focuses on ensuring health and well-being for all has led to increased advocacy and investment in family planning programs [3, 4]. Despite the benefits associated with modern contraceptives, mCPR in sub-Saharan Africa (SSA) remains low [5] hence increasing the risks of negative health outcomes among women [6–8]. Countries like Malawi, Lesotho, Kenya, and Sierra Leone, among others, showed the largest increases in modern contraceptive use between 2010 and 2019 [5] consequently, serving as exemplars of best practices for family planning programs.

Rwanda is one of the countries in SSA that has successfully increased modern contraceptive use [5], rising from 17% in 2005 to 52% in 2015 [9, 10] with significant increases observed among women from rural areas and those with low levels of education [11]. These achievements have been credited to various government-led structural reforms and strategies to drive modern contractive use. These include provincial and administrative decentralization reforms, health sector improvements, involvement of non-governmental partners, performance based financing of health facilities, systematic training of healthcare providers, increased availability and access to modern contraceptives, and enhanced community mobilization and education [4, 11]. In addition, Rwanda launched the National Family Planning and Adolescent Sexual and Reproductive Health (FP/ASRH) and Maternal New-born and Child Health (MNCH) strategic plans (2018–2024) to provide intentional direction for addressing contraceptive and reproductive health challenges for all Rwandans [12].

To promote economic development and enhance healthy lives for Rwandans, the government through the FP/ASRH and MNCH strategic plans (2018–2024) and commitment to the International Conference on Population and Development (ICPD) 25, has articulated the need to promote family planning and “attain zero unmet needs for family planning”. To achieve this, identifying factors that influence change in contraceptive use remains essential in sustaining the gains that have been achieved. Although much of the success in modern contraceptive use has been attributed to structural reforms, [4], understanding changes in population structure dynamics, and personal and sociodemographic factors remain vital in sustaining the gains in modern contraceptive use in Rwanda.

To guide policymakers and planners, the availability of modern contraceptive use information relating to sexually active women is key in sustaining gains in maternal and reproductive health; thus, crucial in measuring progress. Earlier studies conducted in Rwanda [11] and elsewhere [13, 14] employed decomposition analyses to determine factors that influence change in modern contraceptive use among married women. Although the contextual requirements of women differ depending on their marital status [15], achieving SDG 5 which focuses on achieving gender equality and empowering all women and girls depends on access to reproductive health services including contraceptives for all sexually active women, especially teenagers and adolescents among who the prevalence of unwanted pregnancies remains high [16, 17]. In 2010, UNFPA proposed that measuring the progress of modern contraceptive use based only on women who are married or in a union does not present a precise measure as this excludes sexually active adolescents or single women [18, 19] whose sexual activities increase their risks of unintended pregnancies. Several studies from sub-Saharan Africa reported a high prevalence of pregnancies among unmarried teenagers and adolescents [16, 17, 20–22] indicating that sexual activities are not limited to people in union. Based on this, the current study sought to determine and assess determinants of modern contraceptive use among non-pregnant sexually active women in Rwanda.

## Methods

### Study setting

Rwanda is a low-income country with a population size of 12,089,720 [23, 24] but was projected to increase to 12,955,736 in 2021 [25] while its landmass is 26,340 km^2^. The country is densely populated with most of the population concentrated in rural areas [23]. Health care operates on a decentralizing management system; that is, healthcare offices at the district level operate as autonomous entities to provide health care to people within defined zones while specific health programs remain to be operated under a hierarchical management structure [26]. According to the Ministry of Health (MoH) guidelines, modern contraceptives can be provided and accessed at all levels of health care including Health centres/Polyclinics and Dispensary/Clinic/Health posts [26]. In Rwanda, the government subsidizes family planning services, thus these services are offered for free in public clinics operated by the Ministry of Health. Some of the methods offered include contraceptive pills and injections (combined or progestin-only or both), implants, intrauterine devices (IUDs), condoms, spermicides, or diaphragm [12, 26].

### Data

**Fig. 1:**
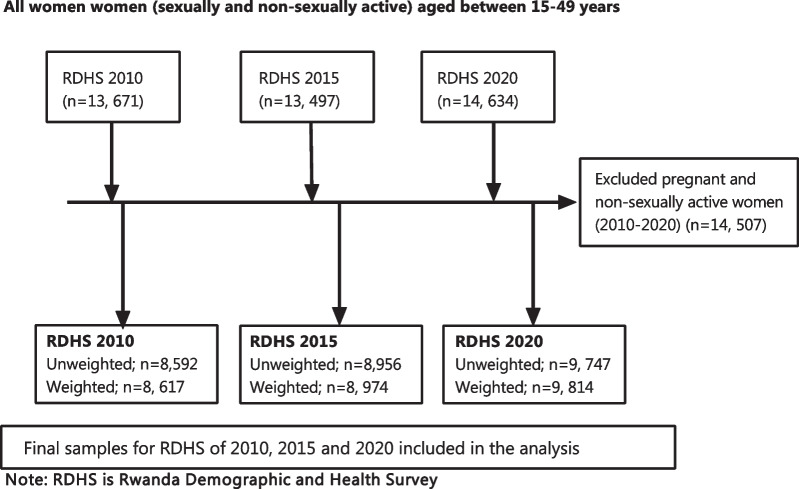
Sampling and inclusion criteria for the number of women included in the analysis from the Rwanda Demographic and Health Survey (RDHS) cycles of 2010, 2015, and 2020 cycles

The current study used secondary data from the Rwanda Demographic and Health Survey (RDHS), collected through nationally representative cross-sectional surveys conducted in 2010, 2015, and 2020 cycles and accessed from the DHS program official database [27]. The RDHS collected demographic, socio-economic, and health data from a representative sample of 13,671 women aged 15–49 years in 12,540 households in 2010, 13,497 women from 12,793 households in 2015 and 14,634 women from 13,000 households in 2020. Rwanda has been stratified into five geopolitical provinces. Each province is made up of enumeration areas (EAs) which then comprise the sampling frame. Respondents included in the DHS were selected using a two-stage sampling strategy. The first stage involved selecting clusters which were the primary sampling units; sampling was done with a probability proportional to the size and the second stage involved a systematic sampling of households from the clusters that had been selected. In the current study, the analysis was focused on sexually active women who at the time of the survey had had sexual intercourse before. Our sample sizes from the three RDHS were 8592 women in 2010 (8617 weighted cases), 8956 in 2015 (8974 weighted cases), and 9747 in 2020 (9814 weighted cases) (Fig. 1). All those who reported being pregnant or not sexually active were excluded from the study.

### Outcome variable and covariates

The outcome variable of interest was the current modern contraceptive use. This variable was categorized as a binary with a “Yes/No” response. In this study, modern contraceptive methods were female sterilization, oral contraceptive pill, intrauterine device, injectables, implants, diaphragms, and condoms as guided by DHS and the Rwanda Ministry of Health [12, 27].

The covariates from our study were classified into four categories:

1. Socio-demographic variables included: Age (15–49 years), area of residence (rural, urban), education levels (None, primary, secondary, higher), a partner education level (None, primary, secondary, higher), wealth index (poor, middle rich), employment status (unemployed, employed), and the number of living children (0, 1–2, 3–4, 5+)
2. Fertility preference: For this study, we abstracted family size concordance (both want the same, husband wants more, husband wants less, don’t know).
3. Family planning program exposure: This was categorized as visiting a health facility in the last 12 months, being visited by community family planning workers in the last 12 months, being told about family planning at a health facility, and exposure to family planning messages through the media. These variables were dichotomized as “Yes” and “No”.
4. Family experience: This was defined as child mortality experience and was dichotomized as “Yes” and “No”.

### Ethics and informed consent

Permission to use the data was obtained from the DHS program after the submission of a written request. All datasets used in our analysis did not contain any identifying information. The original Rwanda DHS 2010, 2015, and 2020 biomarker and survey protocols were approved by the Rwanda Biomedical Centre (RBC) and the Research Ethics Review Board of the Center for Disease Control and Prevention (CDC) Atlanta. The RDHS data collection process required informed consent from participants aged 18 and older. For legal minors aged 15–17 years, the DHS protocol required consent from their parents/guardians before seeking assent from the minors.

### Statistical analysis

To understand the trends in modern contraceptive use between the periods 2010–2020, we used trends analysis stratified by various socio-demographic characteristics. Furthermore, subgroup analysis of trends in modern contraceptive use was done with the subgroups being the different regions stratified by age. Changes in modern contraceptive methods use and modern contraceptive methods between the periods 2010–2015, 2015–2020, and 2010–2020 were analysed using descriptive statistics. To determine and identify factors influencing modern contraceptive use between 2010 and 2020, we used the Oaxaca–Blinder decomposition analysis [28, 29]. This method was initially used two understand labour market outcomes among different groups [28, 29]. Lately, this method has become extensively important in understanding health outcomes by stratifying them by socioeconomic class [30]. This technique has also been applied in evaluating contraceptive use across different groups [31, 32]. The changes in modern contraceptive use between 2010 and 2020 in a multivariate decomposition approach were explained by the change in characteristics or structural changes of the groups between the two-time points (endowments) or by the influence that those characteristics have (coefficients) [11, 29]. This is key in policy formulation because the composition of the population and its behaviour towards contraceptive use is important in designing family planning programs. To carry out these analyses, StataSE 17 [33] was used. Because of the sampling methods used in this study, we used the SVY STATA command to control for the clustering effect. All analyses shown were performed on a weighted population after excluding pregnant and non-sexually active women as shown in Fig. 1.

## Results

### Characteristics of the study population

Table 1 shows the socio-demographic characteristics of sexually active women from three RDHS surveys included in the study. There was an increase in the proportion of women aged between 25 and 49 between 2015 and 2020, after having observed a decrease between the period 2010–2015. Among those aged 15–19 and 20–24 years, the increases observed between 2010 and 2015 were not sustained between 2015 and 2020. In terms of residence, between 2010 and 2020, there was a 5.2% reduction in the proportion of women residing in rural areas. Furthermore, a reduction in the proportion of women with no education and primary education was observed while there was a rise in the proportion of those with secondary and higher education. A reduction in the proportion of people exposed to family planning messages through the media (decreasing from 68.1% in 2010 to 54.2% in 2020) and those who were talked to about family planning at a health facility (decreasing from 58% in 2010 to 36.3% in 2020) was observed. In terms of family size concordance, the proportion of husbands who wanted more children increased from 10.5% in 2010 to 14.7% in 2020. Between 2010 and 2015, there was a 0.4% decrease in the proportion of women who reported having visited healthcare facilities. However, between 2015 and 2020, the proportion of women who reported having visited a healthcare facility increased by 2% (Table 1).

**Table 1:** Socio-demographic characteristics of sexually active women included in RDHS 2010, 2015, and 2019/2020

Background characteristics	(N = 8617)	(N = 8974)	(N = 9814)
	DHS 2010 (%)	DHS 2015 (%)	DHS 2020 (%)
*Age*			
15–19	4.5	5.6	5.0
20–24	14.8	15.0	13.8
25–49	80.7	79.4	81.2
*Residence*			
Urban	14.7	19.6	19.9
Rural	85.3	80.4	80.1
*Education level*			
None	20.8	16.5	12.6
Primary	67.3	68.1	63.6
Secondary	10.2	12.7	19.2
Higher	1.6	2.6	4.5
*Partners education level*			
None	21.8	19.2	13.9
Primary	65.4	68.1	67.5
Secondary	10.8	9.4	13.4
Higher	2.0	3.2	5.2
*Wealth status*			
Poor	41	41.1	39.9
Middle	19.5	19.2	18.6
Rich	39.4	39.7	41.5
*Employment status*			
Unemployed	10.3	7.6	17.2
Employed	89.7	92.4	82.8
*Living children*			
0	9.6	10.6	11.9
1–2	36.6	40.0	38.4
3–4	39.9	38.8	40.0
5+	13.9	10.6	9.7
*Children ever born*			
0	8.7	9.9	11.3
1–2	32.4	37.0	36.0
3–4	25.5	25.9	29.2
5+	33.4	27.2	23.5
*Family size concordance*			
Both want same	58.5	60.4	58.3
Husband wants more	10.5	12.2	14.7
Husband wants less	17.6	17.7	19.7
Don’t know	13.4	9.7	7.3
*Visited health facility in last 12 months*			
No	33.6	34.0	32.0
Yes	66.4	66.0	68.0
*Told about FP at health facility*			
No	42.0	57.6	63.7
Yes	58.0	42.4	36.3
*Visited by a community health worker*			
No	72.5	74.8	70.3
Yes	27.5	25.2	29.7
*Exposure to media FP messages*			
No	31.9	46.4	45.8
Yes	68.1	53.6	54.2

### Trends in modern contraceptive use among sexually active women

**Fig. 2:**
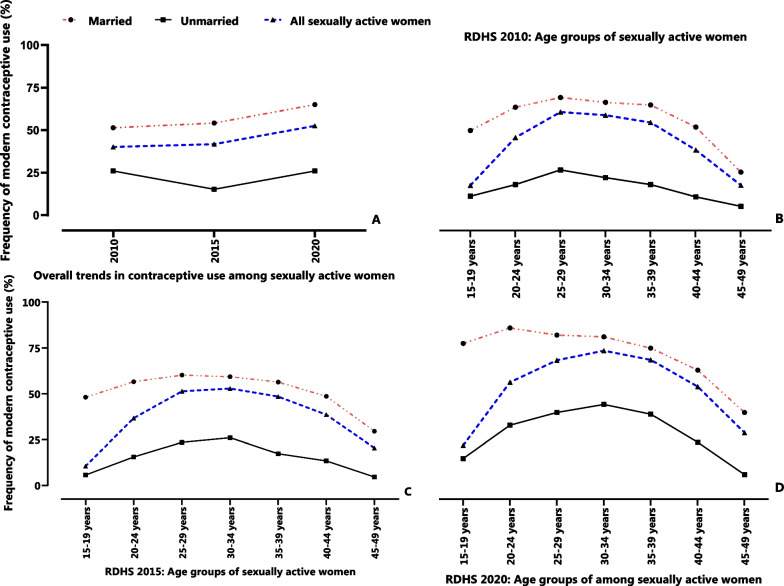
Modern contraceptive use among sexually active women **A** Overall trends in contraceptive use in 2010, 2015, and 2020; **B** modern contraceptive use among women in different age groups in 2010; **C** modern contraceptive use among women in different age groups in 2015; **D** modern contraceptive use among women in different age groups in 2020

Overall, an increase in modern contraceptive use was observed among all women in Rwanda (Fig. 2A). Subgroup stratification showed that between 2010 and 2020, there was an increase in modern contraceptive use among married women. On the other hand, a 10.8% (from 25.9% in 2010 to 15.1% in 2015) decrease in contraceptive use among unmarried women was observed between 2010 and 2015. However, between 2015 and 2020, there was a 10.8% increase in modern contraceptive use. Furthermore, stratification of modern contraceptive use by age indicated a 15.7% overall increase among women aged 30–34 years (Fig. 2B and D). In 2010, 51.9% of all women aged 25–29 years were using modern contraceptives. In 2015 and 2020, 52.9% and 66.0% respectively, of all women aged 30–34 years were using modern contraceptives. The results also indicate a general reduction in the proportion of use of modern contraceptive beyond the age group of 30–34 years (Fig. 2B–D). In addition, the proportion of married women using contraceptives remains relatively higher than that of unmarried women. Between 2010 and 2020, there was a 27% increase in modern contraceptive use among married women aged 15–19 years. This was closely followed by those aged 20–25 years whose overall increase was 22% in the same period (Fig. 2).

**Fig. 3:**
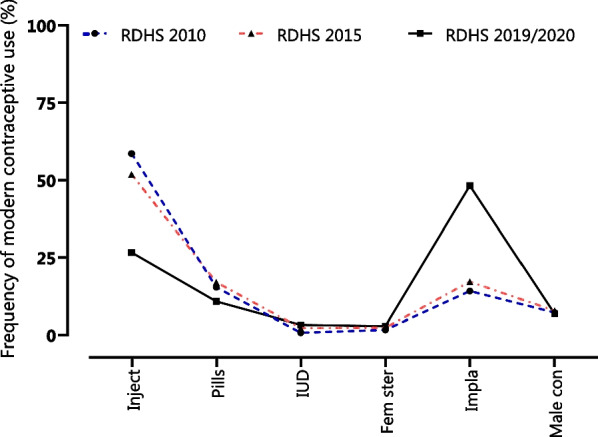
Changes in the preference for modern contraceptive methods among sexually active women in Rwanda between 2010 and 2020. (*Inject* injections, *pill* oral contraceptive pill, *IUD* intrauterine device, *Fem ster* female sterilization, *Impla* implants, male con male condoms)

Although injections were the most used modern contraceptives, among all sexually active women in 2010 and 2015, implants were observed to have been the most preferred modern contraceptive method between 2015 and 2020 (Fig. 3).

The overall point change in modern contraceptive use period between 2010 and 2020 was 12.4%. From 2010 to 2015, there was a 1.7% point change while between 2015 and 2019/2020, there was a 10.5% point change in modern contraceptive use (Table 2). The study showed a 13.1% point increase in modern contraceptive use among women aged 25–49 years old while there was only a 4.4% point change among young women and adolescents aged 15–19 years. Furthermore, there was a 13.9% point increase in modern contraceptive use among women from rural areas. Women with no education and those with primary education levels showed a 14.8% and 12.3% change in modern contraceptive use, respectively. Regarding family size concordance, there was an 18.2% point change among sexually active women who didn’t know. Other changes in modern contraceptive use were observed among women who had not been exposed to media family planning messages, increasing from 4.9% in 2010–2015 to 13.6% between 2015 and 2020 (Table 2).

**Table 2:** Trend in modern contraceptive use among active women15–49 years by selected characteristics, 2010, 2015, and 2019/2020 RDHS

Background characteristics	DHS 2010 (N = 8617)		DHS 2015 (N = 8974)		DHS 2020 (N = 9814)		Percentage point difference in modern contraceptive use (2015–2010)	Percentage point difference in modern contraceptive use (2020–2015)	Percentage point difference in modern contraceptive use (2020–2010)
	Freq	%	Freq	%	Freq	%			
*Age*									
15–19	55	14.8	52	10.6	100	19.2	− 4.2	8.6	4.4
20–24	493	40.0	469	36.7	670	50.4	− 3.3	13.7	10.4
25–49	2912	41.6	3191	44.9	4311	54.7	3.3	9.8	13.1
*Residence*									
Urban	496	39.1	706	40.2	883	45.3	1.1	5.1	6.2
Rural	2954	40.2	3038	42.1	4255	54.1	1.9	12.0	13.9
*Education level*									
None	546	30.4	532	35.8	560	45.2	5.4	9.4	14.8
Primary	2477	42.7	2695	44.1	3433	55	1.4	10.9	12.3
Secondary	356	40.5	418	36.5	928	49.2	− 4	12.7	8.7
Higher	68	48.1	104	44.4	215	48.2	− 3.7	3.8	0.1
*Partners education level*									
None	557	34.4	572	40.0	579	62.8	5.6	22.8	28.4
Primary	2269	46.7	2517	49.6	2941	65.6	2.9	16.0	18.9
Secondary	384	47.9	346	49.2	589	66.3	1.3	17.1	18.4
Higher	78	51.4	117	48.3	204	58.5	− 3.1	10.2	7.1
*Wealth status*									
Poor	1245	35.2	1476	40.0	2129	54.4	4.8	14.4	19.2
Middle	713	42.4	764	44.3	1046	57.3	1.9	13.0	14.9
Rich	1492	43.9	1506	42.3	1964	48.2	− 1.6	5.9	4.3
*Employment status*									
Unemployed	286	32.9	196	29.5	751	46	− 3.4	16.5	13.1
Employed	3060	40.5	3416	42.4	4222	53.6	1.9	11.2	13.1
*Living children*									
0	31	3.7	18	1.9	67	5.7	− 1.8	3.8	2.0
1–2	1372	43.5	1647	45.9	2237	59.4	2.4	13.5	15.9
3–4	1595	46.4	1691	48.5	2397	61.1	2.1	12.6	14.7
5+	456	38	391	41.1	440	46.1	3.1	5.0	8.1
*Children ever born*									
0	27	3.5	15	1.7	63	5.3	− 1.8	3.6	1.8
1–2	1228	43.8	1537	46.5	2084	59.8	2.7	13.3	16.0
3–4	1084	48.9	1177	50.7	1811	64.3	1.8	13.6	15.4
5+	1121	39.1	983	41.3	1123	48.9	2.2	7.6	9.8
*Family size concordance*									
Both want same	1926	55	1998	55.2	2500	65.9	0.2	10.7	10.9
Husband wants more	287	45.7	360	49.2	586	61.1	3.5	11.9	15.4
Husband wants less	591	56.1	590	55.6	854	66.6	− 0.5	11.0	10.5
Don’t know	242	30.3	261	44.8	229	48.5	14.5	3.7	18.2
*Visited health facility in last 12 months*									
No	2269	36.3	2558	38.1	3368	48.8	1.8	10.7	12.5
Yes	1178	49.8	1191	52.7	1774	60.9	2.9	8.2	11.1
*Told about FP at health facility*									
No	802	33.4	1358	39.8	2240	52.7	6.4	12.9	19.3
Yes	1744	52.6	1256	50.0	1434	59.2	− 2.6	9.2	6.6
*Visited by a CHW*									
No	2269	36.3	2558	38.1	3368	48.8	1.8	10.7	12.5
Yes	1178	49.8	1191	52.7	1774	60.9	2.9	8.2	11.1
*Exposure to media FP messages*									
No	918	33.4	1594	38.3	2334	51.9	4.9	13.6	18.5
Yes	2530	43.1	2151	44.7	2807	52.8	1.6	8.1	9.7
Total	3447	40.0	3742	41.7	5143	52.4	1.7	10.7	12.4

Percentage point difference in modern contraceptive use (2015–2010) is the difference between the proportion of people using modern contraceptives under different background characteristics for the year 2015 and 2010. This also applies to the percentage point difference for the years 2020–2015 and 2020–2010

### Factors associated with changes in the use of modern contraceptives

The results of the binary multivariate decomposition regression analysis of factors associated with changes in modern contraceptive use between 2010 and 2020 are presented in Table 3. The results indicated that 23.7% of the overall percentage change in modern contraceptive use was attributable to the difference in women’s characteristics (*compositional changes*). These included level of education, wealth categories, number of living children a woman has, number of children a woman has ever had, access to family planning information from health facilities, and exposure to family planning media messages. An increase in the proportion of women who attained secondary level of education resulted in a significant positive contribution to modern contraceptive use between 2010 and 2020 (7.36%).

**Table 3:** Decomposition of change in modern contraceptive use among sexually active women aged 15–49 years in Rwanda; 2010–2020

Background characteristics	Due to differences in characteristics (E)		Due to differences in coefficients (C)	
	Endowments	%	Coefficients	%
*Age*				
15–19	Ref		Ref	
20–24	− 0.00481	− 4.71	0.01437	14.05
25–49	− 0.00786	− 7.69	− 0.06709	− 65.57
*Residence*				
Urban	Ref		Ref	
Rural	− 0.00008	− 0.08	0.01258	12.29
*Education level*				
None	Ref		Ref	
Primary	− 0.00404**	− 3.95	0.02317	22.64
Secondary	0.00753*	7.36	− 0.00003	− 0.03
Higher	− 0.00051	− 0.49	− 0.00083	− 0.81
*Partners education level*				
None	Ref		Ref	
Primary	0.00013	0.13	− 0.02681	− 26.21
Secondary	− 0.00144	− 1.41	− 0.00527	− 5.15
Higher	− 0.00226	− 2.21	− 0.00028	− 0.28
*Wealth status*				
Poor	Ref		Ref	
Middle	− 0.00041	− 0.40	− 0.01611**	− 15.75
Rich	0.00062**	0.60	− 0.05339***	− 52.18
*Employment status*				
Employed	Ref		Ref	
Unemployed	− 0.00231	− 2.26	− 0.03483	− 34.05
*Living children*				
0	Ref		Ref	
1–2	0.00874***	8.54	0.03103	30.33
3–4	− 0.00199*	− 1.95	0.01082	10.57
5+	− 0.00554	− 5.41	0.0015	1.47
*Children ever born*				
0	Ref		Ref	
1–2	− 0.01279**	− 12.50	− 0.03046	− 29.78
3–4	0.00481***	4.71	− 0.01828	− 17.87
5+	0.03379***	33.03	− 0.01998	− 19.53
*Family size concordance*				
Both want same	Ref		Ref	
Husband wants more	− 0.00200	− 1.96	0.00242	2.37
Husband wants less	0.00000	0.00	− 0.00203	− 1.99
*Told about FP at health facility*				
No	Ref		Ref	
Yes	0.01172*	11.45	− 0.09178***	− 89.71
*Visited by a community health worker*				
No	Ref		Ref	
Yes	0.0016*	1.57	0.00984	9.62
*Exposure to media FP messages*				
No	Ref		Ref	
Yes	− 0.00144	− 1.40	− 0.01918	− 18.75
*Constant*			0.35865**	
*Total*	0.02429***	23.74	0.07801***	76.26

*p < 0.05

**p < 0.01

***p < 0.001

Similarly, an increase in the proportion of women who belonged to households that were categorized as rich led to a significant positive contribution to changes in the use of modern contribution (0.6%). An increase in the proportion of women with a smaller number of living children and a reduction in the proportion of women who reported 5 or more children ever born made a huge contribution to modern contraception of 8.54% and 33.03%, respectively. Accessing family planning information from health facilities also accounted for positive changes in modern contraceptive use between 2010 and 2020. There was a significant increase in the proportion of women who reported having been told about family planning at the health facility during the 2020 survey leading to a positive contribution of 11.45% in modern contraceptive use among sexually active women. Visits by community health workers accounted for an increase in the use of modern contraceptives by 1.57%.

After controlling for all compositional factors included in Table 3, most (76.26%) of the increase in modern contraceptive use was attributed to changes in the effects of coefficients (women’s contraceptive behaviour). Changes in contraceptive behaviour of women with a primary level of education (22.64%) and those with one to two living children (30.33%) contributed the most to modern contraceptive use improvement. These factors showed a significant effect on the observed positive change in modern contraceptive use between 2010 and 2020 surveys. Furthermore, 14.1% of the increase in modern contraceptive use during the study period was contributed by changes in contraceptive behaviour of young women in the age range (20–24 years) while changes in attitude towards modern contraceptive use among women living in rural areas accounted for 12.3% of changes in modern contraceptive use in Rwanda.

## Discussion

This study used a multivariate decomposition analysis to assess modern contraceptive use changes in the past decade among sexually active women in Rwanda. Our results show that modern contraceptive use has been on the rise in Rwanda thus helping the country remain on course of attaining its set family planning goals. Rwanda remains one of the three countries in SSA that have successfully implemented modern contraceptive use programs due to the involvement of partner non-governmental organizations and the government’s commitment to improving the health care system and the health and well-being of its citizens [4, 11]. Of significance to this outcome is that the use of modern contraceptives would enhance the attainment of health equity and improve sexual reproductive health among sexually active women.

In the current study, 23.7% of the change in modern contraceptive use was due to differences in compositional changes such as education, the number of children ever born, and receipt of family planning messages through health facilities. Accessing family planning messages from health facilities contributed the most to the change in modern contraceptive use. Health facilities have been observed to be the primary source of family planning messages [34] owing to the perceived authenticity and credibility of information received [35]. With most of the population residing in rural areas, health care delivery whose coverage in Rwanda is high, plays an essential role in the promotion of modern contraceptive use. In addition, the proportion of women with at least secondary education and its influence on modern contraceptive use corroborate the findings of Cleland and Wilson [36], Emina et al*.* [37], Larsson and Stanfors [38], and Beguy et al*.* [39]. These authors suggested that education can influence modern contraceptive use through economic endowments, information acquisition, and utilization. Educated women are more likely to be informed about different contraceptive methods available, thus making their decisions based on their acquired knowledge. Our results suggest that the promotion of female education remains key in increasing modern contraceptive use. With the available working systems in Rwanda, this can be broadened through the promotion of universal secondary education to include social development activities and enhanced health financing [37].

The current study observed that the age of sexually active women had a negative contribution to the change in modern contraceptive use, an observation that was also made in an earlier study [11]. Our results contrast the outcome of other studies from within East Africa which observed positive contributions [14, 40]. We are of the view that increased awareness and government programs towards promoting modern contraceptive use among women of reproductive age have led to an increase in knowledge and understanding about contraceptive use. This increased awareness has also been observed and reported elsewhere [41]. Furthermore, the availability of modern contraceptive methods and decentralization of access points besides health centres suggests that awareness and knowledge about modern contraceptive use in Rwanda remain high.

Our findings from the decomposition analysis suggest that the overall increase in modern contraceptive use due to behaviour is consistent with the ongoing government agenda of promoting family planning in Rwanda as described in the FP/ASRH Strategic Plan (2018–2024) [12]. Our findings corroborate previous findings by Babalola and Oyenubi [13], Yussuf et al*.* [40]. The current study suggests that a rise in contraceptive use among youths aged 20–24 years and enhanced community visits by community health workers (CHW) may have positively influenced modern contraceptive use. Furthermore, the use of youth-friendly digital family planning and sexual reproductive health learning platforms such as *CyberRwanda* [42], systematic training of community health workers to support family planning at the community level [43], and the creation of an enabling environment to make sexual and reproductive health services responsive to sexually active youths have hastened modern contraceptive uptake and access thus, serving as a lesson to other African countries.

In consonant with the previous study [11], the current study shows that an increase in modern contraceptive use was due to changes in modern contraceptive use among women from rural areas. Improvement of health care delivery among the larger rural population remains essential in attaining health equity. Pivotal to this rise in modern contraceptive use is the community-based health insurance (CBHI) also known as *mutuelles de santé* which provides improved access to health services including reproductive health services. Furthermore, extending access to modern contraceptives through capacity building of government-supported community health workers has led to improved contraceptive uptake [43]. We also observed that a 30.33% and 10.57% increase in modern contraceptive use was due to changes in contraceptive use among women with 1–2 and 3–4 children. Although a large family size has been believed to be ideal in several SSA countries [44], changes in life desires, goals, and aspirations among the younger generation may lead to shifts in the desired family size.

This current study builds on the strengths and observations from an earlier study by Muhoza et al*.* [11] to provide an opportunity to understand modern contraceptive use among sexually active women of reproductive age in Rwanda. To “explore and develop new strategies for reaching more Rwandan of reproductive age” [12], understanding factors that positively influence modern contraceptive use among all women will provide policymakers with the much-needed information to strengthen the existing family planning programs and provide appropriate policies. The current study highlights important compositional and behavioural factors that have driven modern contraceptive use upward in Rwanda. However, the importance of other relatively common variables such as health insurance, number of sexual partners, religion, and socio-cultural and contextual factors could not be determined. This may be regarded as a limitation of this study thus requiring exploration in the future. Furthermore, the cross-sectional nature of RDHS suggests that we could not draw conclusive relationships between modern contraceptive use and the identified determinants of the observed change.

## Conclusion

Rwanda has made great strides in modern contraceptive uptake and sustaining these gains will be vital in attaining vision 2030 and SDGs 3 and 5. The observed changes in modern contraceptive use were attributed to increasing contraceptive uptake among youths aged 20–24 years. Residence, increasing education levels, number of living children, and visitations by a CHW Programme interventions with an emphasis on these variables will be key in enhancing modern contraceptive use in Rwanda. To sustain and enhance the gains made in modern contraceptive use in Rwanda, there is need to encourage multisectoral collaboration in delivering reproductive health programs. Furthermore, increasing focus on reproductive health behaviour change will be key in improving and enhancing knowledge and awareness about modern contraceptives. Also, campaigns to get more sexually active adolescents and youths involved will lead to having a critical mass of informed youths and reducing the risks of unwanted pregnancies.

## Data Availability

Data analysed in this study is publicly available on DHS program website at (https://dhsprogram.com/).
